# Effectiveness of Vitamin E in Treatment of Antipsychotic-Induced Tardive Dyskinesia and Extrapyramidal Symptoms: A Case Report

**DOI:** 10.7759/cureus.68231

**Published:** 2024-08-30

**Authors:** Muhanad Elnoor, Syed Ali Bokhari, Meghana Singh, Idriss A Mohamed

**Affiliations:** 1 Psychiatry, Al Amal Psychiatric Hospital, Emirates Health Services, Dubai, ARE

**Keywords:** psychotic disorder, psychosis, epse, eps, td, extrapyramidal symptoms, tardive dyskinesia, antipsychotic, vitamin e

## Abstract

Antipsychotic medications, while crucial in managing severe psychiatric disorders such as schizophrenia and bipolar disorder, are frequently associated with extrapyramidal symptoms (EPS) and tardive dyskinesia (TD). TD, characterized by repetitive, involuntary movements, especially of the face and limbs, poses a substantial clinical challenge due to its often irreversible nature. Conventional management strategies, including dose reduction and switching to atypical antipsychotics, frequently offer limited success, prompting exploration of alternative therapies. This case report highlights the effectiveness of vitamin E, a potent antioxidant, in treating a 28-year-old male with severe antipsychotic-induced EPS and TD, unresponsive to traditional therapies. The patient, who had been receiving paliperidone injections as part of his psychotic disorder treatment regimen, developed marked EPS, including muscle rigidity, a parkinsonian gait, significant motor disturbances as well as tardive dyskinesia. Despite discontinuation of paliperidone and initiation of procyclidine, propranolol, clonazepam, and omega-3 supplements, his symptoms persisted. Introduction of oral vitamin E at 400 IU daily led to a dramatic improvement, with an 80% reduction in EPS and TD symptoms within weeks. The patient's Abnormal Involuntary Movement Scale (AIMS) score decreased from 24 to 4, and his overall quality of life improved significantly. Gradual increase of vitamin E dosage to 1200 IU daily, coupled with tapering of other medications, eventually led to complete resolution of symptoms, as evidenced by an AIMS score of 0. The patient maintained symptom-free status during follow-up, with no recurrence of psychotic symptoms. This case underscores the potential role of vitamin E as a viable adjunctive treatment for TD, particularly in patients who do not respond adequately to conventional therapies. While the literature presents mixed evidence regarding vitamin E’s effectiveness, this case adds to the growing body of research suggesting its benefits, especially when introduced early in the disease course. Further large-scale studies are warranted to establish the most effective treatment protocols and identify patient populations most likely to benefit from vitamin E therapy.

## Introduction

Antipsychotic medications have revolutionized the treatment of severe psychiatric disorders such as schizophrenia and bipolar disorder. However, their use is often accompanied by significant adverse effects, most notably extrapyramidal symptoms (EPS), which include acute dystonia, parkinsonism, akathisia, and tardive dyskinesia (TD) [1,2]. TD, characterized by repetitive, involuntary movements, particularly of the face, tongue, and limbs, remains a particularly challenging and often irreversible complication associated with prolonged use of these drugs, particularly first-generation antipsychotics, although also seen with second-generation antipsychotics (SGA) [3]. The pathophysiology of TD is complex, involving dopamine receptor hypersensitivity, oxidative stress, and neurotoxic damage, particularly in the basal ganglia [4]. 

Traditionally, management of TD and other EPS has relied on dose reduction or switching to atypical antipsychotics, alongside the use of anticholinergics, benzodiazepines, and other agents, albeit with limited success [5]. In light of this, alternative treatments such as vitamin E, known for its potent antioxidant properties, have gained interest. Vitamin E is hypothesized to mitigate the oxidative damage implicated in TD, and its therapeutic potential has been the focus of several studies [2,6]. Despite some conflicting findings, vitamin E has shown promise, particularly in reducing TD symptoms when administered early [7]. The aim of this case report is to highlight the effectiveness of vitamin E in treating EPS and especially TD, in a patient resistant to conventional therapies, thereby contributing to clinical awareness and potentially expanding treatment options for these debilitating conditions [8].

## Case presentation

**Figure 1:**
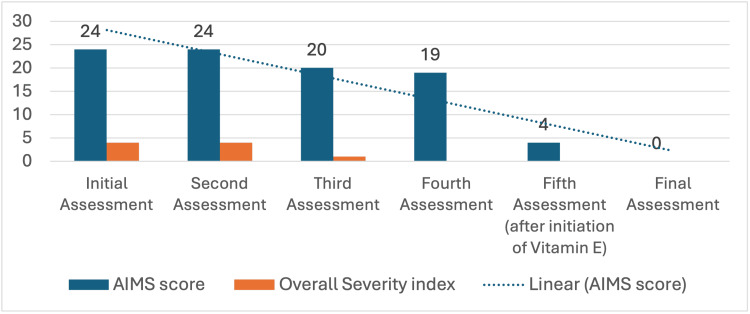
Changes in the Abnormal Involuntary Movement Scale (AIMS) scores over time.

A 28-year-old Middle Eastern gentleman presented to the Emergency department with significant EPS several weeks following the administration of the intramuscular three-monthly paliperidone 350 mg injection as part of his treatment regimen for a psychotic disorder. These EPS symptoms included muscle rigidity, tremors, a parkinsonian gait, drooling, clenched jaw, masked face and jerky limb (robotic) movements. The Abnormal Involuntary Movement Scale (AIMS) score was 24 (Figure 1).

The patient reported that his psychotic symptoms appeared for the first time approximately seven months prior, a month after which he was diagnosed with a psychotic disorder at a different facility. These symptoms included auditory hallucinations, specifically voices making derogatory remarks directed at him, and persecutory delusions, where he believed that family members were practicing witchcraft against him.

He has a longstanding history of polysubstance misuse, primarily involving alcohol and inhalants, as well as behavioural disturbances characterized by aggressive outbursts, particularly when under the influence of alcohol and legal troubles, including multiple incarcerations due to violent behaviour. Notably, these psychotic symptoms were absent during periods of abstinence from substance use. Despite these challenges, he had no history of mood swings, significant depressive episodes, or self-harm.

Upon his first visit to the general adult psychiatric outpatient clinic two weeks after his visit to the Emergency Department (second assessment), he presented with severe EPS, likely induced by the three-monthly paliperidone injection he had received, which had recently been changed from the monthly intramuscular paliperidone 100 mg injections. He denied any history of movement disorders while he was on the monthly paliperidone injection. His symptoms included marked restlessness, muscle rigidity, a parkinsonian gait characterized by poverty of movements including diminished arm swing, tremors, and significant motor side effects such as masked face, drooling, jerky limb movements, and involuntary, fine, rhythmic motions of the mouth along a vertical plane, without involvement of the tongue (known as rabbit syndrome). Additionally, he complained of acute constipation and sleep disturbances, both attributable to the side effects of the medication. Despite these pronounced motor symptoms, his speech remained coherent and relevant, though it was monotonic and reduced in quantity, and his affect was noted to be constricted. His AIMS score was 24 (Figure 1).

Given the severity of his EPS, the initial management plan involved discontinuing the paliperidone injection. He was prescribed procyclidine 5 mg twice daily to manage the EPS, along with propranolol 10 mg twice daily and clonazepam 0.5 mg twice daily, with a gradual tapering of clonazepam planned following the resolution of EPS symptoms. Furthermore, omega-3 supplements were introduced as part of his treatment regimen. He was advised to continue with this treatment plan and scheduled for a follow-up visit. 

Upon subsequent review (third assessment), approximately two weeks later, he exhibited some improvement in his EPS symptoms, with the AIMS score being 20. Nonetheless, the motor side effects, including robotic movements and early signs of TD, persisted. Laboratory investigations revealed an elevated prolactin level and a reduction in white blood cell counts, although no other significant abnormalities were identified. A brain MRI performed as part of his workup was unremarkable. He denied any psychotic symptoms or mood disturbances during this visit and reported that his sleep and appetite were satisfactory. Given the partial improvement in his EPS symptoms, the treatment regimen was maintained without any significant changes.

Approximately one month later, he returned for another follow-up (fourth assessment). At this visit, he continued to report persistent EPS, with an AIMS score of 19, particularly muscle rigidity and a parkinsonian-like gait, along with severe constipation. The clinical examination also revealed the presence of tardive dyskinesia, characterized by involuntary movements, including piano-like finger movements and vermicular movements of the tongue. Despite these ongoing symptoms, he did not report any psychotic or mood disturbances and maintained a euthymic mood. To address the persistent EPS and TD, the treatment plan was adjusted. He was started on vitamin E oral capsules 400 IU once daily, given the emerging evidence of its potential benefits in ameliorating TD symptoms. The remainder of his medication regimen was continued as previously prescribed.

Several weeks later, he presented for another follow-up visit (fifth assessment), reporting a significant improvement in his symptoms. He noted an approximately 80% reduction in the severity of his EPS and TD symptoms, including marked decreases in tremors, muscle rigidity, akathisia, and constipation. His AIMS score had dramatically reduced to 4 (Figure 1). He expressed enthusiasm for engaging in physical exercise, and both his quality of life and social interactions had improved considerably. He continued to deny any psychotic symptoms, mood swings, or abnormal behaviours. As a result of his positive response to the treatment, the plan was adjusted to further optimize his care by gradually increasing the dose of vitamin E to 1200 IU daily. The dose of procyclidine was reduced to 5 mg once daily and then stopped.

During his most recent follow-up visit, he reported a complete resolution of his EPS and TD symptoms. His AIMS score was 0 (Figure 1). He described leading a normal life, returning to his premorbid self, with no new symptoms or side effects, and his sleep and overall well-being had returned to normal. Both he and his family expressed satisfaction with the outcome of his treatment. As a result, his medications were gradually tapered and eventually discontinued, while regular follow-up was continued to monitor for any recurrence of symptoms. Based on his clinical course and the resolution of symptoms following the discontinuation of paliperidone, his diagnosis was revised to drug-induced psychotic disorder rather than schizophrenia. Ongoing psychoeducation sessions were provided to reinforce the importance of avoiding substance abuse to prevent future relapses.

## Discussion

The management of TD and other EPS induced by antipsychotic medications continues to pose a significant challenge in psychiatric practice [5]. TD is not only distressing for patients but also complicates ongoing treatment regimens, often necessitating dose reductions or changes in medication that can compromise the control of underlying psychiatric conditions [1]. Understanding the pathophysiology of TD is crucial in exploring new treatment avenues. The disorder is believed to result from a combination of dopamine receptor supersensitivity and oxidative stress, leading to neurotoxic damage in the brain's motor circuits [3,4]. This pathophysiological framework underpins the rationale for using antioxidants like vitamin E as a therapeutic intervention [6,9].

Vitamin E, a lipophilic antioxidant, has been explored as a potential treatment for TD due to its ability to neutralize free radicals, thereby protecting neurons from oxidative damage [10]. Several studies have demonstrated its efficacy, particularly in cases where TD has a relatively short duration. For example, Lohr et al. (1996) conducted a double-blind, placebo-controlled study that found significant reductions in TD symptoms in patients treated with vitamin E doses of 800 mg oral capsules twice daily, particularly those with a shorter duration of TD [7]. This finding is consistent with the hypothesis that early intervention with antioxidants can mitigate the neurodegenerative processes underlying TD [2].

Further supporting evidence comes from studies like that of Bischot et al. (1993), which reviewed the potential benefits of vitamin E (given up to 1,600 IU per day) in treating extrapyramidal disorders, including TD and Parkinson's disease [6]. The study highlighted that vitamin E's effectiveness might be most pronounced in patients with less chronic forms of TD, where oxidative damage has not yet caused irreversible neuronal injury [9]. Similarly, Dorfman-Etrog et al. (1999) observed a trend toward reduced neuroleptic-induced parkinsonism with vitamin E supplementation, although the effects on other forms of EPS were less clear [4].

In addition to these findings, Post et al. (2002) provided compelling molecular evidence supporting the neuroprotective effects of vitamin E. Their study demonstrated that vitamin E (even with a dose of just 100 IU per day) could attenuate the oxidative stress and apoptosis induced by haloperidol, a common first-generation antipsychotic, thereby preserving neuronal integrity and reducing EPS severity [3]. Moreover, clinical observations have reported that even atypical antipsychotics, such as clozapine, which are generally considered to have a lower risk of EPS, can still induce these symptoms under certain conditions. A case study highlighted that the administration of vitamin E, 400 IU three times daily, significantly ameliorated clozapine-induced extrapyramidal symptoms, further supporting its potential role as a protective agent across different types of antipsychotic-induced motor side effects [11]. These studies collectively suggest that vitamin E could be a valuable adjunctive therapy in managing TD, particularly when initiated early in the disease course [10].

Despite the promising results, the literature presents a mixed picture regarding the overall efficacy of vitamin E in treating TD. The Cochrane Systematic Review (2020) analyzed data from multiple randomized controlled trials and found that while vitamin E may prevent the worsening of TD symptoms, its ability to significantly improve existing symptoms was limited [10]. This conclusion was drawn from studies like those by Eranti et al. (1998), which found no protective effect of vitamin E against acute EPS induced by haloperidol [12], and Lazzarini et al. (2005), which even reported that vitamin E might exacerbate cataleptic symptoms when combined with certain drugs [9]. 

Moreover, a large multicenter study failed to demonstrate significant benefits of vitamin E over placebo in treating TD, raising questions about its overall efficacy in more chronic cases of the disorder [5]. These findings suggest that while vitamin E may have a role in preventing the progression of TD, particularly in the early stages, it may not be effective as a standalone treatment for more established cases [8,10]. This variability in clinical outcomes could be due to differences in study design, patient populations, or the duration and dosage of vitamin E treatment [4,6].

Given the mixed evidence, the use of vitamin E in clinical practice requires careful consideration [2]. It appears that vitamin E may offer the greatest benefit when used as part of a broader treatment strategy, particularly in patients with early-stage TD or those at risk of developing EPS [1,13]. Its favourable safety profile makes it an attractive option for adjunctive therapy, but clinicians should be mindful of its limitations and the potential for adverse effects when combined with certain neuroleptics [9,14].

The findings from our case report, where vitamin E significantly improved TD symptoms in a patient who had not responded to conventional treatments, suggest that vitamin E could be a valuable addition to the therapeutic arsenal for managing TD and EPS [7,10]. This case adds to the growing body of evidence that supports the use of antioxidants such as vitamin E in treating neuroleptic-induced movement disorders, emphasizing the need for further research to determine which patients are most likely to benefit [5,6]. 

Future research should focus on large-scale, well-designed clinical trials to better understand the circumstances under which vitamin E is most effective [2,10]. Additionally, exploring the combination of vitamin E with other antioxidant therapies or neuroprotective agents could provide insights into more comprehensive treatment strategies for TD and other EPS [3,12]. Understanding the genetic and biochemical factors that predispose certain patients to benefit from vitamin E could also pave the way for more personalized treatment approaches in the future [9,13].

## Conclusions

This case highlights the potential efficacy of vitamin E as an effective therapy for managing TD and other EPS, particularly when initiated early in the course of the disorder. Despite mixed results in the literature, our case report supports its role in improving patient outcomes where conventional treatments have failed. Further research is necessary to fully understand the optimal application of vitamin E and to identify which patient populations will benefit most from its use. 
